# Social capital as a determinant of quality of life in greek cancer patients: a cross-sectional study

**DOI:** 10.3389/fpubh.2025.1602804

**Published:** 2025-08-15

**Authors:** Athanasios Pitis, Maria Diamantopoulou, Aspasia Panagiotou, Dimitrios Papageorgiou, Foteini Tzavella

**Affiliations:** Department of Nursing, School of Health Sciences, University of Peloponnese, Tripoli, Greece

**Keywords:** social capital, quality of life, cancer, social determinants of health, nursing

## Abstract

**Introduction:**

Health equity continues to be an obstacle in Greece. The EU Cancer Inequalities Registry indicates that social determinants of health significantly influence the cancer burden in Greece, thus affecting quality of life.

**Methods:**

This was a cross-sectional study; the Social Capital Questionnaire Greek version was used for the evaluation of individual social capital, and the EORTC Core Quality of Life questionnaire (EORTC QLQ-C30) was used for the assessment of quality of life. The Kolmogorov–Smirnov test was used for checking the normality distribution of the quantitative variables. Quantitative variables were expressed as mean values (Standard Deviation) and as median (interquartile range), while categorical variables were expressed as absolute and relative frequencies. The association between QoL and Social capital scales was checked via Spearman correlation coefficients (rho).

**Results:**

Greater total score in Social capital scale was significantly associated with greater overall QoL (*β* = 0.005; *p* < 0.001), better Emotional functioning (*β* = 0.004; *p* = 0.004) and better Social functioning (*β* = 0.009; *p* = 0.002),with lower Nausea and vomiting (*β* = −0.017; *p* = 0.015), Insomnia (*β* = −0.014; *p* = 0.002), Appetite loss (*β* = −0.010; *p* = 0.018) and Constipation symptoms (*β* = −0.009; *p* = 0.047).

**Conclusion:**

There was a strong association between a greater social capital score and the quality of life of Greek cancer patients, indicating that higher levels of social capital are associated with improved quality of life.

## 1 Introduction

According to the Global Cancer Observatory, in 2022, 65.703 patients had been diagnosed with cancer in Greece, and 32,385 individuals lost their lives to the disease the same year (1). Health equity remains a challenge in Greece, connected to the financial crisis that escalated in 2009 and to the distinctive sociodemographic characteristics of the country’s population. Access to healthcare, including characteristics such as accessibility to medical facilities, physician-to-population ratio, and availability of cancer detection technologies and screening procedures, constitutes essential elements of socioeconomic hardship. The EU Cancer Inequalities Registry indicates that social determinants of health significantly influence the cancer burden in Greece (2). Between 2010 and 2020, the country’s lifetime cancer prevalence grew by 26%, compared to 24% in the EU. As cancer patients live longer and more of them have a history of the disease, it is crucial to prioritize quality of life and survivorship (3).

The World Health Organization (WHO) defines quality of life (QoL) as “an individual’s perception of their position in life within the context of the culture and value systems in which they exist, and in relation to their goals, expectations, standards, and concerns,” (4) whereas “health-related quality of life” (HRQoL) emphasizes QoL in terms of health-related factors. Nonetheless, HRQoL is an extensive and complex word lacking a universally accepted meaning (5); existing definitions differ between those that prioritize physical, social, and emotional well-being and those that highlight the impact of an individual’s health on daily life (6).

“Social determinants of health broadly defined as the conditions in which people are born, grow, live, work and age, and people’s access to power, money and resources” influence health outcomes (7). People’s relationships and the social systems in which they live have an impact on their health (8, 9). Among these determinants, one particularly important is social capital. The concept of social capital encapsulates the significance of social connections to health and well-being. In order to understand how social links make lives more meaningful, sociologists, economists, and social theorists have worked on the concept of social capital for almost a century (10). “Social capital has many different conceptualizations (10–12), but “most scholars agree that it pertains to civic engagement, density of social networks, information channels, shared values, trust, mutual support, and reciprocity among people. The fundamental principle is that social interactions and engagement in community activities are investments that promote individual and collective well-being” (13). Research on social capital has also shown connections between health and quality of life, life expectancy, and health behaviors, as well as neighborhood characteristics (14).

The relevance of these conceptual frameworks becomes particularly evident in oncology, where the interplay between social disadvantages and disease is both complex and consequential. Patients suffering from cancer often express a need for assistance due to the negative impact social determinants of health may have on them (15, 16). Recently the pandemic has led to a sudden and absolute mode of action by governments through the horizontal imposition of social distancing as a means of defense against COVID-19. This interference in personal choices has had immediate short-term and long-term consequences, escalating existing challenges such as immediate access to healthcare, preventive check-ups, follow-ups, unemployment and poverty (17), leading patients to exhibit increased health-related social needs such as poverty, food instabilities, transportation issues and housing insecurities (18, 19).

All these health-related social constraints, along with the social loss frequently experienced by cancer patients because of alienation (20), may prevent access use and timely delivery of effective oncology care leading to negative cancer outcomes (16, 18, 19, 21–25). Research indicates that health-related social demands and especially lower social capital are linked to disparities in cancer prevalence, severity, accessibility to treatment, and disease outcomes (18, 19) that can lead to lower quality of life, greater recurrence rates, and lower survival rates (26–29).

Because of the increased social needs Greek cancer patients face we hypothesized that greater social capital would lead to better quality of life. The purpose of this study was to evaluate the association between social capital and quality of life in Greek cancer patients undergoing treatment.

## 2 Materials and methods

### 2.1 Participants

This is a multicenter cross-sectional study that was conducted from April 2023 to April 2024 in General Hospital of Athens Ippokrateio, Athens General Hospital of Thoracic Diseases Sotiria, Theageneio Anticancer Hospital of Thessaloniki and General Oncology Hospital of Kifisia Agioi Anargiroi. General Hospital of Athens Ippokrateio and Athens General Hospital of Thoracic Diseases Sotiria, are both tertiary-level healthcare institutions that provide services to the population of Attica region and operate specialized oncology units, Theageneio Anticancer Hospital of Thessaloniki is one of the four tertiary-level oncology hospitals across Greece that provides cancer care services to the population of the wider region of Macedonia and patients from northern Greek islands, General Oncology Hospital of Kifisia Agioi Anargiroi is one of the four tertiary-level oncology hospitals across Greece that provides cancer care services to the population of the wider region of Attica and also provides cancer care to patients from Greek islands. Patients that participated in the study were recruited from the Chemotherapy Day Units of the hospitals while undergoing anticancer therapy. Patients were recruited during active anticancer therapy at chemotherapy day units in each hospital. To be included, patients had to satisfy the following inclusion criteria: be adult patients, be fluent in Greek, have been undergoing treatment for a minimum of 4 weeks at the time of the survey, be diagnosed with solid tumors, and be capable of providing informed consent. Individuals with hematological malignancies were excluded, as were individuals with cognitive and behavioral impairments that may have limited their ability to complete the evaluation tools. A total of 400 questionnaires were returned.

### 2.2 Measurements

#### 2.2.1 Sociodemographic and clinical characteristics

A questionnaire was developed to collect information on sociodemographic and clinical factors such as gender, educational level, family status, economic situation, cancer type, metastasis, cancer stage, and therapy type(s). The kind of malignancy, metastatic illness, and type(s) of treatment were determined by reviewing the patients’ files. The economic status was assessed using a 5-point Likert scale.

#### 2.2.2 Social capital

For the assessment of social capital, the validated Greek version of the Social Capital Questionnaire (SCQ-G) was used, which is a self-completed tool. The “Social Capital Questionnaire” was developed in Australia (30). It consists of 36 questions, which are divided into eight factors measuring different dimensions of the term “social capital” in the original version, and six in the Greek version. The axes cover the concepts: “Participation in the local community,” “Feelings of safety,” “Family and friends connections,” “Tolerance of diversity,” “Value of life and social agency,” “Work connections.” Participants are asked to answer the questions based on a 4-point Likert scale. In all questions, a higher score on the scale is an indication of higher social capital. Scoring is done either for the scale as a whole or for the individual factors that make up the scale. The psychometric validation of SCQ in a Greek sample demonstrated its validity and applicability as a measure of individual-level social capital in Greece (13, 31).

#### 2.2.3 Quality of life

For the assessment of quality of life, the validated Greek version of EORTC Core Quality of Life questionnaire (EORTC QLQ-C30) was used. The questionnaire is self-administered, and the questions evaluate the patient’s status during the last week. The instrument comprises 30 items, that evaluate five functional dimensions (physical, social, emotional, cognitive, and role functioning), three symptom dimensions (fatigue, pain, nausea, and vomiting), six individual symptom items evaluating additional cancer-related symptoms (dyspnoea, insomnia, appetite loss, constipation, diarrhea, and economic impact) along with treatment, and a global health status/QoL scale. The first 28 items can be answered in 4-point Likert-type scale, with the following format: “not at all,” “a little,” “quite a bit,” and “very much.” Items 29 and 30 are global scales that include seven possible answers, ranging from 1—“very poor” to 7—“excellent.” Higher scores on the symptom scale signify a decrease in QoL due to cancer-related symptoms, while higher values on the global and functional health scales indicate better QoL. The questionnaire was well accepted in the Greek population and had very high reliability (32).

### 2.3 Statistical analysis

The Kolmogorov–Smirnov test was used to check the normality distribution of the quantitative variables. Since the assumption of normality was not satisfied for all under study scales, non-parametric tests or specific transformations were used for their analysis. Quantitative variables were expressed as mean values (Standard Deviation) and as median (interquartile range), while categorical variables were expressed as absolute and relative frequencies. The association between QoL and Social capital scales was checked via Spearman correlations coefficients (rho). The coefficient is considered very high when it is above 0.9, high when it is 0.7–0.9, moderate when it is 0.5–0.7, low when it is 0.3–0.5 and very low when it is below 0.3 (33). The associations between QoL and Social capital scales were also examined, after adjusting for all patients’ characteristics, via multiple linear regression analysis with dependent variables the QoL subscales. The regression equation included terms for patients’ demo-graphical and clinical characteristics, as well as their scores in Social capital scale one at a time, due to high intercorrelation. Adjusted regression coefficients (*β*) with standard errors (SE) were computed from the results of the linear regression analyses. Logarithmic transformations of the QoL scales were used for the regression analyses. Internal consistency reliability was determined by the calculation of Cronbach’s alpha coefficient. Scales with reliabilities equal to or greater than 0.70 were considered acceptable. Missing data were handled by listwise deletion in the analysis. All reported *p* values are two-tailed. Statistical significance was set at *p* < 0.05 and analyses were conducted using SPSS statistical software (version 27.0).

## 3 Results

### 3.1 Sociodemographic and clinical factors

Four hundred cancer patients participated in the study, with mean age being 61.7 years (SD = 12.5 years). Their characteristics are presented in Table 1. Most patients were females (50.8%), high school graduates (30.5%), married (60.0%), with children (79.3%) and moderately satisfied from their economic status (51.1%). Moreover, 27.5% had been diagnosed with lung cancer and 23% with gastrointestinal Cancer. Metastatic cancer had 45.5% of the sample and 45.3% had stage IV diagnosis.

**Table 1:** Sample’s characteristics.

*n* = 400	Social and clinical factors	*n* (%)
Demographical characteristics	Gender	
	Males	197 (49.3)
	Females	203 (50.8)
	Age, mean (SD)	61.7 (12.5)
	Educational level	
	Primary school	71 (17.8)
	Middle school	46 (11.5)
	High school	122 (30.5)
	Technical university	46 (11.5)
	University	73 (18.3)
	MSc	33 (8.3)
	PhD	9 (2.3)
	Family status	
	Unmarried	54 (13.5)
	Married	240 (60.0)
	Civil partnership	6 (1.5)
	Divorced	48 (12.0)
	Separated	6 (1.5)
	Widowed	46 (11.5)
	Children	317 (79.3)
	Satisfaction from economical status	
	Not at all	62 (15.7)
	A little	52 (13.2)
	Moderately	202 (51.1)
	Much	67 (17.0)
	Very much	12 (3.0)
Clinical characteristics	Type of cancer	
	Gastrointestinal Cancers	92 (23.0)
	Head &Neck Cancers	7 (1.8)
	Sarcomas	19 (4.8)
	Gynecological Cancers	22 (5.5)
	Lung Cancer	110 (27.5)
	Breast cancer	89 (22.3)
	Bladder cancer	20 (5.0)
	Pancreatic cancer	29 (7.3)
	Other	12 (3.0)
	Metastatic	182 (45.5)
	Disease stage	
	Ι	30 (7.5)
	ΙΙ	95 (23.8)
	ΙΙΙ	94 (23.5)
	IV	181 (45.3)
	Surgery	240 (60.0)
	Chemotherapy	399 (99.8)
	Radiotherapy	92 (23.0)
	Immunotherapy	139 (34.8)

### 3.2 Descriptive measures and reliability indexes

Descriptive measures and reliability indexes of QoL and social capital scales are presented in Table 2. Mean Global health status score was 59.35% (SD = 22.02%) and mean total social capital score was 71.93 (SD = 10.94). Cronbach’s alpha coefficients were above 0.7 in all subscales, indicating acceptable reliability.

**Table 2:** Descriptive measures and reliability indexes of QoL and social capital scales.

QoL and social capital subscales	Minimum	Maximum	Mean (SD)	Median (IQR)	P Kolmogorov–Smirnov test	Cronbach’s alpha
Global health status/QoL^1^	0.00	100.00	59.35 (22.02)	58.33 (50.00 ─ 75.00)	<0.001	0.80
Fatigue^2^	0.00	100.00	46.69 (29.37)	44.44 (22.22 ─ 66.67)	<0.001	0.83
Nausea and vomiting^2^	0.00	100.00	9.63 (19.63)	0.00 (0.00 ─ 16.67)	<0.001	0.70
Pain^2^	0.00	100.00	26.82 (30.91)	16.67 (0.00 ─ 50.00)	<0.001	0.87
Dyspnoea^2^	0.00	100.00	34.83 (33.17)	33.33 (0.00 ─ 66.67)	<0.001	–
Insomnia^2^	0.00	100.00	36.25 (36.86)	33.33 (0.00 ─ 66.67)	<0.001	–
Appetite loss^2^	0.00	100.00	19.75 (31.33)	0.00 (0.00 ─ 33.33)	<0.001	–
Constipation^2^	0.00	100.00	21.00 (32.07)	0.00 (0.00 ─ 33.33)	<0.001	–
Diarrhoea^2^	0.00	100.00	17.75 (29.83)	0.00 (0.00 ─ 33.33)	<0.001	–
Financial difficulties^2^	0.00	100.00	35.58 (34.25)	33.33 (0.00 ─ 66.67)	<0.001	–
Physical functioning^1^	0.00	100.00	66.93 (22.31)	66.67 (53.33 ─ 86.67)	<0.001	0.80
Role functioning^1^	0.00	100.00	59.21 (32.50)	66.67 (33.33 ─ 83.33)	<0.001	0.81
Emotional functioning^1^	0.00	100.00	69.48 (24.35)	75.00 (58.33 ─ 91.67)	<0.001	0.71
Cognitive functioning^1^	0.00	100.00	81.42 (23.93)	83.33 (66.67 ─ 100.00)	<0.001	0.54
Social functioning^1^	0.00	100.00	61.38 (32.13)	66.67 (33.33 ─ 91.67)	<0.001	0.79
Participation in the local community^3^	12.00	42.00	20.15 (5.06)	19.00 (16.00 ─ 24.00)	<0.001	0.71
Feelings of safety^3^	2.00	8.00	5.45 (1.59)	6.00 (4.00 ─ 6.00)	<0.001	0.75
Family and friends connections^3^	2.00	8.00	4.16 (1.28)	4.00 (3.00 ─ 5.00)	<0.001	0.72
Tolerance of diversity^3^	2.00	8.00	4.50 (1.56)	4.00 (3.00 ─ 6.00)	<0.001	0.75
Value of life and social agency^3^	15.00	44.00	33.00 (5.46)	34.00 (29.00 ─ 37.00)	<0.001	0.77
Work connections^3^	4.00	16.00	11.12 (2.71)	11.00 (9.00 ─ 13.00)	0.004	0.76
Social Capital total score^3^	43.00	104.00	71.93 (10.94)	71.00 (64.00 ─ 80.00)	0.012	0.82

In scales with one item, Cronbach’s alpha coefficient could not be computed.

^1^greater values indicate better QoL; ^2^greater values indicate worse QoL; ^3^greater values indicate higher social capital.

### 3.3 Correlational analysis

Spearman correlation coefficients (rho) among QoL and Social capital subscales are presented in Table 3. In most cases, there were significant correlation coefficients among QoL and Social capital subscales, in a way that greater social capital was associated with less symptoms and greater overall QoL and functioning. From multiple linear regression analysis, after adjusting for all patients’ characteristics, emerged that greater score in subscale “Feelings of safety” was significantly associated with greater overall QoL (*β* = 0.018; *p* = 0.030) and less fatigue (*β* = −0.043; *p* = 0.021) and insomnia (*β* = −0.106; *p* < 0.001) symptoms. Greater score in subscale “Family and friends connections” was significantly associated with greater overall QoL (*β* = 0.024; *p* = 0.032) and less fatigue symptoms (*β* = −0.058; *p* = 0.017). Moreover, greater score in subscale “Tolerance of diversity” was significantly associated with greater overall QoL (*β* = 0.018; *p* = 0.044) and less fatigue (*β* = −0.055; *p* = 0.017) and pain (*β* = −0.055; *p* = 0.050) symptoms. Greater score in “Value of life and social agency” subscale was significantly associated with greater overall QoL (*β* = 0.012; *p* < 0.001), better Emotional functioning (*β* = 0.012; *p* < 0.001) and better Social functioning (*β* = 0.022; *p* < 0.001). On the other hand, greater score in “Value of life and social agency” subscale was significantly associated with lower Nausea and vomiting (*β* = −0.017; *p* = 0.015), Insomnia (*β* = −0.036; *p* < 0.001), Appetite loss (*β* = −0.034; *p* < 0.001) and Financial difficulties (*β* = −0.029; *p* < 0.001). Greater score in subscale “Work connections” was significantly associated with greater overall QoL (*β* = 0.016; *p* = 0.002), better emotional functioning (*β* = 0.014; *p* = 0.024) and less insomnia (*β* = −0.095; *p* = 0.003). Overall, greater total score in Social capital scale was significantly associated with greater overall QoL (*β* = 0.005; *p* < 0.001), better Emotional functioning (*β* = 0.004; *p* = 0.004) and better Social functioning (*β* = 0.009; *p* = 0.002). On the other hand, greater total score in Social capital scale was significantly associated with lower Nausea and vomiting (*β* = −0.017; *p* = 0.015), Insomnia (*β* = −0.014; *p* = 0.002), Appetite loss (*β* = −0.010; *p* = 0.018) and Constipation symptoms (*β* = −0.009; *p* = 0.047). According to R^2^ indexes, the percentage of variance explained ranged from 0 to 16%. The greater values were found in Global health status/QoL, where 16% of its variance was explained by participants’ characteristics and their score on “Work connections” subscale and in Financial difficulties, where 14% of its variance was explained by participants’ characteristics and their score on “Value of life and social agency” dimension subscale Table 4.

**Table 3:** Spearman correlation coefficients (rho) among QoL and Social capital subscales.

QoL and social capital subscales	Spearman correlation coefficients (rho)	Participation in the local community	Feelings of safety	Family and friends connections	Tolerance of diversity	Value of life and social agency	Work connections	Social Capital total score
Global health status/QoL	rho	0.16	0.19	0.20	0.14	0.35	0.25	0.29
	P	0.002	<0.001	<0.001	0.006	<0.001	0.010	<0.001
Fatigue	rho	−0.07	−0.11	−0.12	−0.10	−0.20	−0.05	−0.17
	P	0.175	0.027	0.018	0.050	<0.001	0.620	0.001
Nausea and vomiting	rho	−0.07	−0.07	0.02	−0.10	−0.08	−0.04	−0.09
	P	0.180	0.148	0.638	0.044	0.102	0.688	0.076
Pain	rho	0.10	−0.08	0.03	−0.10	−0.08	0.04	−0.03
	P	0.070	0.105	0.506	0.048	0.095	0.652	0.584
Dyspnoea	rho	0.01	−0.08	0.07	−0.05	−0.10	0.07	−0.05
	P	0.864	0.133	0.146	0.335	0.046	0.482	0.366
Insomnia	rho	−0.05	−0.21	−0.09	−0.05	−0.21	−0.28	−0.18
	P	0.277	<0.001	0.061	0.278	<0.001	0.003	<0.001
Appetite loss	rho	−0.03	0.00	−0.08	−0.04	−0.20	−0.12	−0.14
	P	0.530	0.932	0.099	0.382	<0.001	0.200	0.005
Constipation	rho	−0.10	−0.01	−0.08	−0.07	−0.10	0.05	−0.11
	P	0.049	0.885	0.120	0.145	0.048	0.590	0.026
Diarrhea	rho	0.06	−0.05	0.02	0.02	−0.04	−0.08	0.02
	P	0.203	0.346	0.690	0.764	0.429	0.425	0.755
Financial difficulties	rho	−0.04	−0.17	−0.12	−0.08	−0.28	−0.14	−0.23
	P	0.451	0.001	0.021	0.095	<0.001	0.162	<0.001
Physical functioning	rho	0.15	0.14	0.15	0.15	0.21	0.07	0.20
	P	0.003	0.006	0.003	0.003	<0.001	0.475	<0.001
Role functioning	rho	−0.01	0.06	0.04	0.08	0.09	0.13	0.05
	P	0.816	0.217	0.406	0.099	0.089	0.177	0.282
Emotional functioning	rho	0.03	0.11	0.06	0.12	0.27	0.29	0.20
	P	0.617	0.022	0.246	0.015	<0.001	0.003	<0.001
Cognitive functioning	rho	0.08	0.07	0.11	0.10	0.18	0.02	0.14
	P	0.126	0.193	0.023	0.045	<0.001	0.816	0.004
Social functioning	rho	0.09	0.07	0.08	0.07	0.19	0.21	0.16
	P	0.071	0.182	0.114	0.149	<0.001	0.031	0.001

This analysis was univariate, i.e., not adjustment for patients’ demographics was made.

**Table 4:** Results from multiple regression analyses with QoL scores as dependent variables and social capital scores as independent variables, adjusted for patients’ characteristics.

Dependent variables	Independent variables:																				
	Participation in the local community			Feelings of safety			Family and friends connections			Tolerance of diversity			Value of life and social agency			Work connections			Social Capital total score		
	β (SE)+	*p*	*R* ^2^	β (SE)+	*p*	*R* ^2^	β (SE)+	*p*	*R* ^2^	β (SE)+	*p*	*R* ^2^	β (SE)+	*p*	*R* ^2^	β (SE)+	*p*	*R* ^2^	β (SE)+	*p*	*R* ^2^
Global health status/QoL	0.004 (0.003)	0.114	0.07	0.018 (0.008)	0.030*	0.07	0.024 (0.011)	0.032*	0.07	0.018 (0.009)	0.044*	0.07	0.012 (0.003)	<0.001*	0.11	0.016 (0.005)	0.002*	0.16	0.005 (0.001)	<0.001*	0.10
Fatigue	−0.004 (0.006)	0.474	0.02	−0.043 (0.018)	0.021*	0.03	−0.058 (0.024)	0.017*	0.03	−0.026 (0.019)	0.178	0.02	−0.022 (0.006)	<0.001*	0.06	−0.036 (0.020)	0.082	0.02	−0.009 (0.003)	0.001*	0.04
Nausea and vomiting	−0.009 (0.007)	0.226	0.03	−0.029 (0.022)	0.180	0.03	−0.005 (0.029)	0.853	0.03	−0.055 (0.023)	0.017*	0.04	−0.017 (0.007)	0.015*	0.04	−0.004 (0.026)	0.865	0.00	−0.008 (0.003)	0.026*	0.04
Pain	0.027 (0.019)	0.102	0.05	−0.037 (0.027)	0.167	0.03	0.029 (0.035)	0.414	0.02	−0.055 (0.028)	0.050*	0.03	−0.007 (0.009)	0.433	0.02	−0.002 (0.030)	0.936	0.08	0.003 (0.004)	0.541	0.02
Dyspnoea	0.015 (0.009)	0.092	0.01	−0.029 (0.027)	0.296	0.01	0.070 (0.036)	0.058	0.02	−0.004 (0.029)	0.889	0.01	−0.012 (0.009)	0.188	0.01	0.001 (0.032)	0.982	0.00	0.001 (0.004)	0.782	0.01
Insomnia	−0.002 (0.009)	0.788	0.00	−0.106 (0.028)	<0.001*	0.03	−0.044 (0.037)	0.236	0.00	−0.035 (0.030)	0.239	0.00	−0.036 (0.009)	<0.001*	0.04	−0.095 (0.031)	0.003*	0.06	−0.014 (0.004)	0.002*	0.02
Appetite loss	−0.001 (0.009)	0.894	0.00	−0.004 (0.027)	0.895	0.00	−0.042 (0.036)	0.233	0.00	−0.023 (0.029)	0.412	0.00	−0.034 (0.009)	<0.001*	0.03	−0.029 (0.031)	0.353	0.00	−0.010 (0.004)	0.018*	0.01
Constipation	−0.015 (0.009)	0.095	0.00	−0.001 (0.028)	0.965	0.00	−0.049 (0.036)	0.175	0.00	−0.037 (0.029)	0.204	0.00	−0.016 (0.009)	0.067	0.00	0.000 (0.031)	0.988	0.00	−0.009 (0.004)	0.047*	0.00
Diarrhea	0.014 (0.008)	0.099	0.01	−0.023 (0.026)	0.375	0.01	0.025 (0.034)	0.472	0.01	−0.003 (0.028)	0.922	0.01	−0.011 (0.008)	0.185	0.01	−0.018 (0.031)	0.554	0.03	0.001 (0.004)	0.797	0.01
Financial difficulties	0.015 (0.008)	0.072	0.11	−0.024 (0.026)	0.362	0.11	−0.044 (0.034)	0.194	0.11	−0.024 (0.027)	0.377	0.11	−0.029 (0.008)	<0.001*	0.14	−0.017 (0.030)	0.576	0.08	−0.008 (0.004)	0.065	0.11
Physical functioning	0.002 (0.002)	0.406	0.10	0.009 (0.006)	0.123	0.11	0.009 (0.008)	0.270	0.11	0.008 (0.006)	0.205	0.11	0.003 (0.002)	0.090	0.11	0.002 (0.005)	0.725	0.02	0.002 (0.001)	0.088	0.11
Role functioning	−0.008 (0.006)	0.168	0.02	0.006 (0.018)	0.756	0.01	0.016 (0.024)	0.496	0.01	0.020 (0.019)	0.279	0.02	0.004 (0.006)	0.523	0.01	−0.014 (0.016)	0.366	0.06	−0.001 (0.003)	0.706	0.02
Emotional functioning	0.002 (0.003)	0.489	0.08	0.006 (0.009)	0.511	0.08	0.018 (0.012)	0.155	0.08	0.017 (0.010)	0.095	0.08	0.012 (0.003)	<0.001*	0.11	0.014 (0.006)	0.024*	0.05	0.004 (0.001)	0.004*	0.10
Cognitive functioning	−0.003 (0.003)	0.292	0.03	0.003 (0.009)	0.722	0.03	−0.002 (0.012)	0.868	0.03	0.007 (0.010)	0.487	0.03	0.005 (0.003)	0.117	0.03	−0.001 (0.008)	0.949	0.01	0.000 (0.001)	0.843	0.03
Social functioning	0.010 (0.006)	0.098	0.04	0.006 (0.018)	0.756	0.04	0.030 (0.024)	0.208	0.04	0.032 (0.019)	0.092	0.05	0.022 (0.006)	<0.001*	0.07	0.013 (0.019)	0.471	0.05	0.009 (0.003)	0.002*	0.06

+regression coefficients (Standard Error) adjusted for all patients’ demographical and clinical characteristics.

*indicates statistically significant finding.

## 4 Discussion

Our findings suggest that there is a significant association between greater Social Capital and greater overall QoL. Furthermore, there were significant associations between greater scores in subscales “Feelings of Safety,” “Family and Friends connections,” “Tolerance of Diversity,” “Value of Life and Social Agency,” “Work Connections,” “Overall Social Capital” and greater scores in “Overall QoL.” These findings underline the essential impact of social capital -and in a broader context the importance of social relations and needs- as a determinant of the quality of life of Greek cancer patients. While limited research has examined this issue in Greek cancer populations, there are several studies whose conclusions corroborate ours. A review in palliative care settings highlighted the strong association between social capital and quality of life in people with significant health difficulties. In palliative care facilities, social capital has been identified as a contributing factor influencing well-being and the quality of life in end-of-life care, highlighting how the structuring of social networks can improve access to care and maintain support for patients and caregivers (9). A qualitative study in patients with lower socioeconomic status, receiving palliative care indicate that deficiencies in individual, communal, and civic networks might intensify the difficulties of end-of-life care, thereby impacting patient well-being (34).

Two cross-sectional studies that evaluated the association of social capital in the quality of life of breast cancer survivors (35) and multiple sclerosis patients (36) reported strong correlations between higher individual social capital (evaluated by social involvement, network linkages, or cognitive judgments of support), and improved quality of life outcomes (35, 36). These findings indicated that mediating variables, like physical exercise and stress reduction, may also affect this association by converting the advantages of strong social networks into measurable health enhancements (35).

Similarly, studies that investigated the compliance of patients with cancer screening protocols, revealed that social capital that was characterized by increased trust, reciprocity, and engagement, promoted greater awareness and utilization of preventive services, was linked with early detection and overall patient satisfaction and quality of life (37, 38). A Czech study on the other hand, underscored social capital as a significant predictor of quality of life, with elevated community trust and network integration associated with enhanced life satisfaction (39). A study that had been conducted in Lagos, Nigeria, that evaluated the impact of social capital in breast cancer management, revealed that social relations with family, friends, extended networks, community and religion, has been demonstrated to promote support and improve coping mechanisms and treatment adherence, thus augmenting psychological resilience and enhancing overall quality of life (40). A study that evaluated the association between psychological well-being and social capital in cancer patients indicated that strong support systems can effectively reduce stress and enhance resilience, which are associated with improved quality of life outcomes (41).

The existing research regarding healthcare settings highlights the significant impact social capital has on health outcomes and quality of life. The Rhea study in Greek maternal health settings found that greater individual social capital correlates with improved birth outcomes and reduced postpartum depressive symptoms, indicating that reliable social networks and community involvement during pregnancy may act as protective factors (42, 43). Interventions addressing social needs have surfaced as a possible strategy to enhance these favorable outcomes. Increased social support systems, as demonstrated by studies in populations of breast and bladder cancer patients, mitigate psychological distress, foster improved treatment compliance, and diminish pain levels (44, 45). Moreover, addressing comprehensive social determinants of health, such as transportation, food insecurity, and housing instability, has proven essential in alleviating barriers to care and in diminishing disparities that negatively impact the quality of life for at-risk cancer populations (16).

Our findings emphasize the need for tailored interventions and support for cancer patients to address lower social capital and their holistic social requirements, therefore enhancing their quality of life. The collaboration of a multidisciplinary team is essential in the care of oncological patients to ensure holistic care and to address the patient’s needs across all domains. Moreover, it is essential to examine the social dimensions of quality of life that are affected, and to develop strategies for symptom management in cancer patients. Social needs may be evaluated by social workers, hospital social services, or suitably trained nurses, who spend significant time with cancer patients in daily clinical practice using concise tools that evaluate social determinants of health.

While our findings align with those of previous research and we concluded that social capital is a determinant in the quality of life of Greek cancer patients, our study has limitations. Despite the strengths of the present study, certain limitations related to generalizability must be acknowledged. The sample was derived exclusively from tertiary-level oncology hospitals located in two major urban centers of Greece, Athens and Thessaloniki, which may introduce a regional hospital bias. Patients treated in rural or remote areas, where access to specialized care and social resources may be more limited, were underrepresented. Furthermore, all participants were recruited from chemotherapy day units during active outpatient treatment, thereby excluding individuals receiving inpatient care, those in advanced stages of disease, or patients with significant functional decline who may be less able to access outpatient services. In addition, the inclusion criteria requiring fluency in Greek and cognitive ability to complete self-administered questionnaires may have systematically excluded vulnerable groups, such as immigrants, linguistic minorities, and individuals with cognitive impairments populations that often experience social marginalization and reduced social capital. The self-reported instruments that were used may introduce a potential response bias. All data regarding social capital and quality of life were collected through self-administered instruments which may be subject to desirability bias, recall bias and subjective interpretation of items. These factors should be taken into account when interpreting the findings and may limit the extent to which the results can be generalized to the broader population of cancer patients across Greece. The cross-sectional study design is a significant limitation and limits our ability to draw causal conclusions; future research should aim to clarify the directionality and potential causality of the relationship between social capital and quality of life and also consider assessing social capital both at the onset of treatment and upon completion after several months to more accurately evaluate its impact on quality of life. Additionally, further research could analyze social capital in relation to specific cancer types or treatments or based on varying residential locations and social environments. Future research should explore the creation of nurse-led interventions focused on social support and managing social needs in cancer patients to enhance quality-of-life outcomes. Healthcare professionals, including nurses, social workers, psychologists, and other members of the multidisciplinary team, alongside community leaders and sociologists could play a supportive role in fostering social connections that may enhance patients’ social capital. By employing communication strategies such as active listening, patient education, and motivational interviewing, they may assist individuals in accessing local community resources, senior centers, and patient advocacy groups. Furthermore, structured peer support initiatives and group-based interventions, such as cancer support groups, creative therapeutic activities including art and music therapy, or storytelling circles could contribute to cultivating shared identity, mutual support, and trust among patients. These activities are recognized as potential pathways to strengthen social ties, which are fundamental elements of social capital and have been associated with better health outcomes in the literature (46, 47).

## Data Availability

All relevant data is contained within the article: The original contributions presented in the study are included in the article, further inquiries can be directed to the corresponding author.
